# Remarks on the Treatment of Exposed Pulps and Pulpless Teeth

**Published:** 1894-11

**Authors:** D. D. Atkinson

**Affiliations:** Brunswick, Ga.


AMERICAN JOURNAL

—OF—

DENTAL SCIENCE.

Vol. xxviii. Baltimore, November, 1894. No. 7.

ARTICLE I.

### REMARKS ON THE TREATMENT OF EXPOSED PULPS AND PULPLESS TEETH.

BY D. D. ATKINSON, D. D. S., BRUNSWICK, GA.

Just as the architect must have a sufficient base for the structure he is to erect, so must the dentist be certain that when he has exercised his best skill to make a beautiful filling, or crown or bridge, that the foundation upon which it all depends is equal to the demands which may be made upon it, that is to say, the roots of the teeth so filled must be in a healthy state, and are liable to remain so, so far as operative ingenuity can devise, otherwise no matter how much care he has exercised, nor how great his skill in manipulating gold, he will be subject to the mortification at any time of having his patient return in intense agony from acute alveolar abscess, or of seeing his work extracted by other hands. In treating upon this subject, I beg leave to take it in a practical way, just as we have to deal daily in office practice, and hope hereby to elicit discussion out of which will be evolved ideas beneficial to us all.

This article has nothing to do with conservative treatment or capping of the pulk. We will discuss

Devitalization of the pulp and subsequent treatment.

Putrescent pulp canals.

Filling the canals.

If the pulp be exposed, it may be detected by pressure in the cavity with a pellet of cotton, or often by a lack of sensibility in the dentine, and the operator may freely use the excavator even to the point of exposure without the slightest pain until he presses upon or touches the pulp itself. If there is any sensation at the periphery or other point away from the point of exposure, it is reasonably certain that the pulp is not exposed.

Arsenic is the agent commonly used for the purpose of destroying the pulp. Its application is frequently attended with intense pain a few hours after it has been applied.

An eminent writer has said in substance upon this point, that if he wished to punish any one from a grudge, he would want no better method than to place arsenic in a tooth and then pack in cotton saturated with sandarac varnish to hold it in place. He attributes the pain to the fact that the saturated cotton presses upon the pulp and causes pain in a mechanical way. My observation has led me to a different conclusion. If cotton saturated with sandarac varnish be placed in any cavity where the pulp is intact, though not exposed, the same pain will follow as where the pulp is exposed, although there can be no pressure upon the nerve itself. Alcohol *per se* will cause the same pain; the solvent seems to be the cause of the trouble in so far as the arsenic is not considered. Then we must seek something better than the varnish. We find exactly what is desired in chloropercha, the chloroform being in itself a splendid obtundent, and the gutta percha impervious to moisture, a small pellet of cotton is saturated with it just as with the varnish and placed in the cavity, after first having placed a dry pellet over the arsenic. There is no pain in this operation. In this connection let me say that a pellet of cotton saturated with thick chloro-percha will act as a temporary filling and preserve any cavity perfectly for months. Arsenic is a corrosive poison, and consequently will cause violent pain as soon as it has had sufficient time to act. This may be prevented by incorporating with it sulphate of morphia and carbolic acid at the time the application is made.

We often find that the application has devitalized the body of the pulp while that part in the root canals is left in a very sensitive condition, when, if the canals are large enough, a second application can be made, and the whole removed painlessly; but sometimes these are inaccessible, or the canals very small, and it seems impossible to reach the remaining pulp with the medicine, in which case cocain may be used with more or less success, or the work may be completed with sharp drills, which after all causes considerable pain.

The pulp devitalized, it is good practice to extirpate it at once and drill the root canals as near the apex as possible, using a Gates-Glidden drill as large as the size of the root will admit, having previously cut away enough of the crown to admit of direct access to the root canals, if such a thing is possible. (This is a cardinal point which must always be observed, that alveolar abscess rarely follows pivot or crown work may be attributable to the fact that the crowns are so much cut away as to render the greatest facility for reaching the pulp canal.) Then place a pellet of cotton saturated with absolute alchohol in the cavity and seal with another pellet saturated with thick chloro-percha. After one or two renewals of this treatment the root canal will be ready to be filled.

Of putrescent pulps, we find these some times in teeth which have large metallic fillings resting near the pulp cavity, which by reason of their proximity have caused the death of the pulp, or in teeth which have suffered from traumatic injury, or have large cavities in them. When brought to the observation of the dentist, it may be in a state of pericementitis or of rest; if the former, the treatment is simply to open into the pulp cavity and dismiss the patient for that day; if the latter, the treatment is the same; any more than that would be liable to force septic matter, (which abounds within,) through the apical foramen, and induce violent pericementitis and alveolar abscess. At the second sitting of either case, the treatment is practically the same. Wash the cavities thoroughly with absolute alcohol, leaving a pellet of cotton saturated with it in the cavity, and sealed as above stated with chloro-percha. At the third sitting, wash afresh with alcohol, and drill out the root canals. It will be found that there will be no unpleasant odor from within. If there is any soreness, iodoform used with the alcohol, will be found beneficial. It has been urged that this latter has no therapeutic value except what it derives from the iodine it contains, but since its application is nearly always attended with beneficial results, the presumption must stand that it contains iodine in the proper form for dental uses.

The number of sittings necessary in the treatment of pulpless teeth must of course be left to the judgment of the operator. In writing of alcohol, I do it because I find it best in my practice; in its antiseptic and cleansing properties it is the peer of anything brought to my notice in dental materia medica. It can be used with more freedom and facility and efficiency than anything I have used. The two points essential in treating pulpless teeth are asepticism and closing the apical foramen. For the latter, many ideas of the best means prevail, some of which I will enumerate, but will not discuss except in classes: Filling with gold foil; filling with tin foil; filling with sterilized wood points; filling with lead points; filling with wood points dipped in chloro percha; filling with gutta-percha cones; filling with amalgam; filling with floss silk; filling with cotton; filling with beeswax.

In so far as practice is limited to those materials which are non-absorbent, it seems to be scientific, and is productive of good results, but to place cotton, silk or wood, either sterilized or with chloro-percha, in the root of a tooth seems to me to invite disaster, and an unhappy culmination. They take up whatever effusion of plasma or serum as may come in contact with the apex of the tooth root, and if once separated from the circulation of the blood, undergoes decomposition, forms a centre of infection, which in time causes pericementitis and abscess. I will go into detail only as regards one method of filling roots. Pink gutta-percha is known to be non absorbent, and compatible with tooth structure. It is warmed, and a cone twisted between the fingers and allowed to cool, then the point of it dipped into thin chloro-percha and pressed firmly into the prepared canal, effectually closing the apical foramen and is easily applied.

To those who have not tried these points, I will commend their use, irritation rarely follows it, and if it should, the root filling can be easily removed either with a warm instrument or by dissolving with chloroform and treatment renewed. This department in dental surgery has become so much improved within the past decade that I am happy to say it is possible to save almost any carious tooth to the service of the patient, to the honor of dentistry, and to the emolument of the operator.—Proceedings of Georgia State Dental Association.

